# Daughters at Risk of Female Genital Mutilation: Examining the Determinants of Mothers’ Intentions to Allow Their Daughters to Undergo Female Genital Mutilation

**DOI:** 10.1371/journal.pone.0151630

**Published:** 2016-03-31

**Authors:** Tahereh Pashaei, Koen Ponnet, Maryam Moeeni, Maryam Khazaee-pool, Fereshteh Majlessi

**Affiliations:** 1 Department of Public Health, School of Health, Kurdistan University of Medical Sciences, Sanandaj, Iran; 2 Department of Communication Studies, University of Antwerp, Sint-Jacobsstraat 2, 2000, Antwerp, Belgium; 3 Higher Institute for Family Sciences, Odisee, Huart Hamoirlaan 136, 1030, Brussels, Belgium; 4 Faculty of Law, University of Antwerp, Venusstraat 23, 2000, Antwerp, Belgium; 5 Health Management and Economics Research Center, Isfahan University of Medical Sciences, Isfahan, Iran; 6 Department of Health Education and Promotion, School of Health, Zanjan University of Medical Sciences, Zanjan, Iran; 7 Department of Health Education and Promotion, School of Public Health, Tehran University of Medical Sciences, Tehran, Iran; Queensland University of Technology, AUSTRALIA

## Abstract

Female genital mutilation (FGM) is still a common practice in many countries in Africa and the Middle East. Understanding the determinants of FGM can lead to more active interventions to prevent this harmful practice. The goal of this study is to explore factors associated with FGM behavior among Iranian mothers and their daughters. Based on Ajzen’s theory of planned behavior, we examined the predictive value of attitudes, subjective norms, perceived behavioral control and several socio-demographic variables in relation to mothers’ intentions to mutilate their daughters. A paper-and-pencil survey was conducted among 300 mothers (*mean age* = 33.20, *SD* = 9.09) who had at least one daughter and who lived in Ravansar, a county in Kermanshah Province in Iran. Structural equation modeling was used to investigate the relationships among the study variables. Our results indicate that attitude is the strongest predictor of mothers’ intentions to allow their daughters to undergo FGM, followed by subjective norms. Compared to younger mothers, older mothers have more positive attitudes toward FGM, perceive themselves as having more control over their behavior and demonstrate a greater intention to allow their daughter to undergo FGM. Furthermore, we found that less educated mothers and mothers living in rural areas had more positive attitudes toward FGM and feel more social pressure to allow FGM. The model accounts for 93 percent of the variance in the mothers’ intentions to allow their daughters to undergo FGM. Intervention programs that want to decrease FGM might focus primarily on converting mothers’ neutral or positive feelings toward FGM into negative attitudes and on alleviating the perceived social pressure to mutilate one’s daughter. Based on our findings, we provide recommendations about how to curtail mothers’ intentions to allow their daughters to undergo FGM.

## Introduction

The World Health Organization [1] defines female genital mutilation as any procedure that cuts or harms female genitalia without medical indication. As of 2014, there are about 130 million women living in one of the 29 countries of Africa and the Middle East who have experienced female genital mutilation; it is believed that in the next decade, a further 30 million girls, mostly living in Africa and Asia, are in danger of undergoing this harmful practice [2,3]. Although the rate of FGM is slowly diminishing worldwide, the prevalence of FGM in different parts of the world continues at a rate ranging from 0.6% to 98% [3]. FGM can have serious adverse effects on the physical and mental health of women in both the short and long terms, especially since it is often done in unsanitary conditions by relatives, neighbors and elderly women [4,5]. In the short term, severe pain, hemorrhage and abscess from unsterile instruments are the most common problems associated with female genital mutilation. The long-term physical effects of FGM include recurrent infections, keloids, fistulas, pain during sexual intercourse, menstrual problems and even infertility [4,6]. Furthermore, FGM is often associated with serious psychological and emotional difficulties including anxiety, depression, stress, insomnia, eating disorders and impaired cognition [4,7–9]. Consequently, FGM has been recognized as a violation of human rights [7,10] and is considered a public health problem [11].

FGM is mainly carried out on young girls under the age of 15 years old who often have little choice in the matter [7]. FGM is often deeply embedded in the culture and traditions of the people, which influence mothers to want to have their daughters undergo this surgery [12]. The main reasons given for the practice of FGM are prevalent social norms [13,14], the suppression of female sexuality [2,15–17], aesthetic preferences [13], social cohesion [18] and religion [3,16]. The attempt to stop FGM is not easy [19,20]. Previous studies on the practices of female genital mutilation have concluded that the development and implementation of legislation as a sole strategy against FGM is not an effective way to reduce its prevalence [21,22]. Although laws signal a country’s disapproval of FGM and send a clear message of support to those who abandon the practice [23], their deterrent effect has not been confirmed [24]. For instance, in a systematic review on practices of FGM in seven different African countries, it was found that only study participants from Burkina Faso mentioned legal prohibition as a reason for not performing FGM. Burkina Faso, however, is the only country in which people who break the law are commonly prosecuted [25]. Participants from nearly all other African countries consistently argued that FGM was simply a cultural tradition and, therefore, must continue [24]. A more effective way to reduce FGM involves community education and awareness. Successful community strategies that have been documented include mutilation-free rite-of-passage ceremonies and collective declarations in which villages pledge not to mutilate their daughters [25]. Furthermore, several studies have argued that educational interventions that emphasize the negative consequences of FGM, that correct faulty knowledge or that provide educational knowledge that is missing can trigger changes in beliefs [24]. In these intervention programs, various people should be targeted, including parents, health workers, religious leaders and other key people [14,26].

The present study took place in Ravansar, a county in Kermanshah Province in Iran. A recent study conducted by Pashaei [12] found that the prevalence of FGM in the region is approximately 58% and that 96% of the genital mutilations are performed by traditional midwives and old women. Moreover, the same study revealed that FGM is always done based on a mother’s request. In other words, mothers allow others to genitally mutilate their daughters. This indicates that FGM is deeply embedded in the culture and traditions of the people, which makes the reduction and elimination of the practice of FGM very challenging [4]. Understanding the factors associated with FGM behavior might help in the designing of appropriate intervention strategies to change this behavior. Therefore, the aim of this study is to test a clinically relevant and theoretical framework to explain and predict mother’s intentions to have their daughters undergo FGM; the reason for this approach is that intention is a very strong determinant of actual behavior [27]. One such framework that has proved to be successful is the theory of planned behavior (TPB) [27,28]. To date, TPB is one of the most thoroughly tested and robust of the social psychological models for understanding, predicting and changing health-related behaviors [29–33]. To the best of our knowledge, no study has applied this framework to predict mothers’ intentions to make their daughters undergo female genital mutilation.

The theory of planned behavior (TPB) proposes that behavior can be predicted by the strength of an individual’s intention to behave in a particular way. Behavioral intention is predicted by three variables: attitude toward the behavior, subjective norms and perceived behavioral control over the behavior [28]. Attitude refers to people’s positive or negative evaluation of the behavior. A subjective norm is defined as the perceived social pressure to perform or not to perform the behavior. Perceived behavorial control refers to the level of ease or difficulty the individual experiences when attempting to perform the behavior [27]. As a general rule, the more favorable the attitude and the subjective norm and the greater the perceived behavioral control, the stronger is the person’s intention to engage in the behavior [27,28]. The stronger the person’s intention, the more that person is expected to try, and hence, the greater the likelihood of the person demonstrating the behavior [34]. Except with regard to behaviors that are largely out of the person’s behavioral control, the intention to engage in a particular behavior is the strongest predictor of the actual behavior [28,35]. Based on the TPB-literature, we derive three hypotheses (H): There is a positive association between mothers’ attitudes (H1), subjective norms (H2) and perceived behavioral control (H3) and their intention to allow their daughters to undergo FGM.

In sum, the present study was conducted in Ravansar, a county in which FGM is still a common practice. The purpose of the study was to gain a better understanding of mothers’ intentions to allow their daughters to undergo FGM. We therefore examined the predictive value of attitudes, subjective norms and perceived behavioral control in relation to mothers’ intentions to allow their daughter to undergo FGM. The findings of this study may help policy makers design appropriate interventions to prevent the practice of FGM among the new generation of females in Iran.

## Material and Method

### Procedure and participants

Data were collected with the help of five trained midwives who worked in health centers located in both urban and rural areas of Ravansar, a county in western Iran. Ravansar is a region in Iran in which FGM is practiced fairly commonly [36]. Almost 100% of the population in Ravansar has a Kurdish background (i.e., an ethnic minority in Iran) [36]. To identify potential study subjects, the midwives reviewed the existing medical records of mothers who had been referred to the health centers in 2011. In Iran, each family has a medical file at a health center located in the region in which they live. Mothers were eligible if they (a) had experienced female genital mutilation, (b) had at least one daughter younger than seven years old and (c) had no daughter(s) above seven years old. The age criteria were chosen because a recent study [12] found that approximately 54% of the women in Ravansar are mutilated before they are seven years old. All eligible women (*n* = 323) were called by the midwives and were informed about the purpose of the study. All of the contacted women had a medical record at the health center. Therefore, they were asked by phone to come to the health center. In the correspondence with the participants, the Kurdish language was used because it is the ethnic language of Ravansar and is known by all the citizens in the region.

The midwives were trained by the first author in how to contact the mothers. The trained midwives asked the subjects whether they agreed to participate in the study. It was made clear that the respondents were under no obligation to participate and that their responses would be treated anonymously and confidentially. Furthermore, the mothers were assured that their decision to refuse would not deprive them of any health services at the health centers. Mothers who were willing to participate received an introductory letter explaining the purpose and procedure of the survey. Each participant also received a plain-language statement and a written informed-consent form. If women were illiterate, the midwives helped the mothers to fill in the paper-and-pencil questionnaire. No compensation was given to the respondents. The study protocol was approved by the ethics committee of the Tehran University of Medical Sciences.

### Measures

Following the recommendations of Ajzen [37] and Francis and colleagues [38], a self-report questionnaire was developed to assess the constructs of the theory of planned behavior. The questionnaire was built with the aid of health workers (i.e., experienced midwives) who possessed academic knowledge of FGM and were trained to examine mothers and diagnose whether they had ever been mutilated. In order to check if there were any ambiguities or if the respondents had any difficulty in responding to the items, a pilot study was conducted among 30 women who had been referred to the above-mentioned health centers. The final questionnaire included items on attitudes toward female genital mutilation, subjective norms, perceived behavioral control and mothers’ intentions to mutilate their daughters (measured using the binary question “Do you intend to make your daughter undergo female genital mutilation?”) and the abovementioned socio-demographic variables. A total of 32.2% (*n* = 97) of surveyed mothers intended to do so.

#### Attitude

To measure mothers’ attitudes toward female genital mutilation, the respondents were asked to answer seven items (e.g., “Female genital mutilation is a good tradition”). The items were scored on a 5-point Likert scale ranging from 1 = *strongly disagree* to 5 = *strongly agree*. The reliability of the scale was good (α = .92).

#### Subjective norm

Three items measured the mothers’ own estimates of the social pressure to perform the target behavior (e.g., “Family members expect me to mutilate my daughter”). The items were scored on a 5-point Likert scale ranging from 1 = *strongly disagree* to 5 = *strongly agree*. The internal consistency of the items was good (α = .83).

#### Perceived behavioral control

One item measured women’s confidence that they were capable of preventing their daughters from being genitally mutilated: “Despite the difficulties, I can prevent my daughter from undergoing FGM.” The item was scored on a 5-point Likert scale, ranging from 1 = *strongly disagree* to 5 = *strongly agree*.

Descriptions of the variables are presented in Table 1.

**Table 1:** Descriptions of the variables.

	Mean	Standard Deviation (SD)	Skewness	Kurtosis
**Attitude**				
Att1. Female genital mutilation is a good tradition	2.14	1.27	.64	-.93
Att2. Female genital mutilation is good for controlling female sexuality	2.50	1.04	.49	-.41
Att3. Female genital mutilation is performed for religious reasons	2.10	1.24	.77	-.67
Att4. Female genital mutilation increases women’s health	2.13	1.21	.62	-.98
Att5. Female genital mutilation is a violent behavior (reverse scored)	2.47	1.07	.35	-.57
Att6. Female genital mutilation should continue	2.34	1.14	.37	-.98
Att7. Female genital mutilation increases the chances of marriage	2.52	1.09	.32	-.51
**Subjective norm**				
Sn1. Family members expect me to mutilate my daughter	2.66	1.26	.25	-1.02
Sn2. Neighbors put me under pressure to mutilate my daughter	2.94	1.15	.21	-.89
Sn3. My husband wants me to mutilate my daughter	2.89	1.18	.35	-.89
**Perceived behavioral control**				
Despite the difficulties, I can prevent my daughter from undergoing female genital mutilation	2.33	.81	.60	.28

### Analytic strategy

To investigate the relationships among the study variables, structural equation modeling (SEM) was applied to the collected data using Mplus 6 [39]. The analyses were carried out in the following way. First, we built a measurement model and examined whether the observed variables reliably reflected the hypothesized latent variables in the research model. The latent constructs, i.e., attitudes and subjective norms, were created using the above-mentioned manifest variables. Second, we estimated a structural model with attitudes, subjective norms and perceived behavioral control as predictor variables, and behavioral intention as the endogenous variable. The age and education of the mothers, and whether they lived in an urban or rural area were included as covariates in the model.

Several fit indices were used to evaluate the model fits of the measurement and path models. Given that the χ^2^ is almost always significant and not an adequate test of the model fit [40], we also reported the Comparative Fit Index (CFI), the Root Mean Square Error of Approximation (RMSEA) and the Standardized Root Mean Square Residual (SRMR). The CFI ranges from 0 to 1.00, with a cut-off of .95 or higher indicating that the model provides a good fit and .90 indicating that the model provides an adequate fit [41]. RMSEA values below .05 indicate a good model fit, and values between .06 and .08 indicate an adequate fit [42,43]. An SRMR value below 0.08 indicates a good model fit [41].

## Results

### Sample characteristics

In total, 300 mothers who had at least one daughter who was younger than seven years old and with a history of FGM filled out the questionnaire. The mean age of the mothers was 33.20 years (*SD* = 9.09, skewness = .41). Regarding the place of residence, 49% (*n* = 147) of the women lived in an urban area, and 51% (*n* = 153) lived in a rural area. Education was measured according to the highest level of education the mothers had achieved. Within the sample, 43% (*n* = 129) of the mothers had completed fewer than 6 years of education (primary school), 39.7% (*n* = 119) had completed secondary or high school education and 17.3% (*n* = 50) of the women had completed higher education. In the sample, 93.3% of the mothers (*n* = 280) were married, 3.3% (*n* = 10) were single and 3.3% (*n* = 10) were divorced. With regard to employment, 93.7% of the respondents (*n* = 281) were unemployed, which is comparable to the 90% unemployment rate for women in Kermanshah province [44]. Many mothers (61.7%, *n* = 185) had experienced acute or chronic complications related to FGM as an infant, child or teenager.

### Measurement and structural model

**Fig 1:**
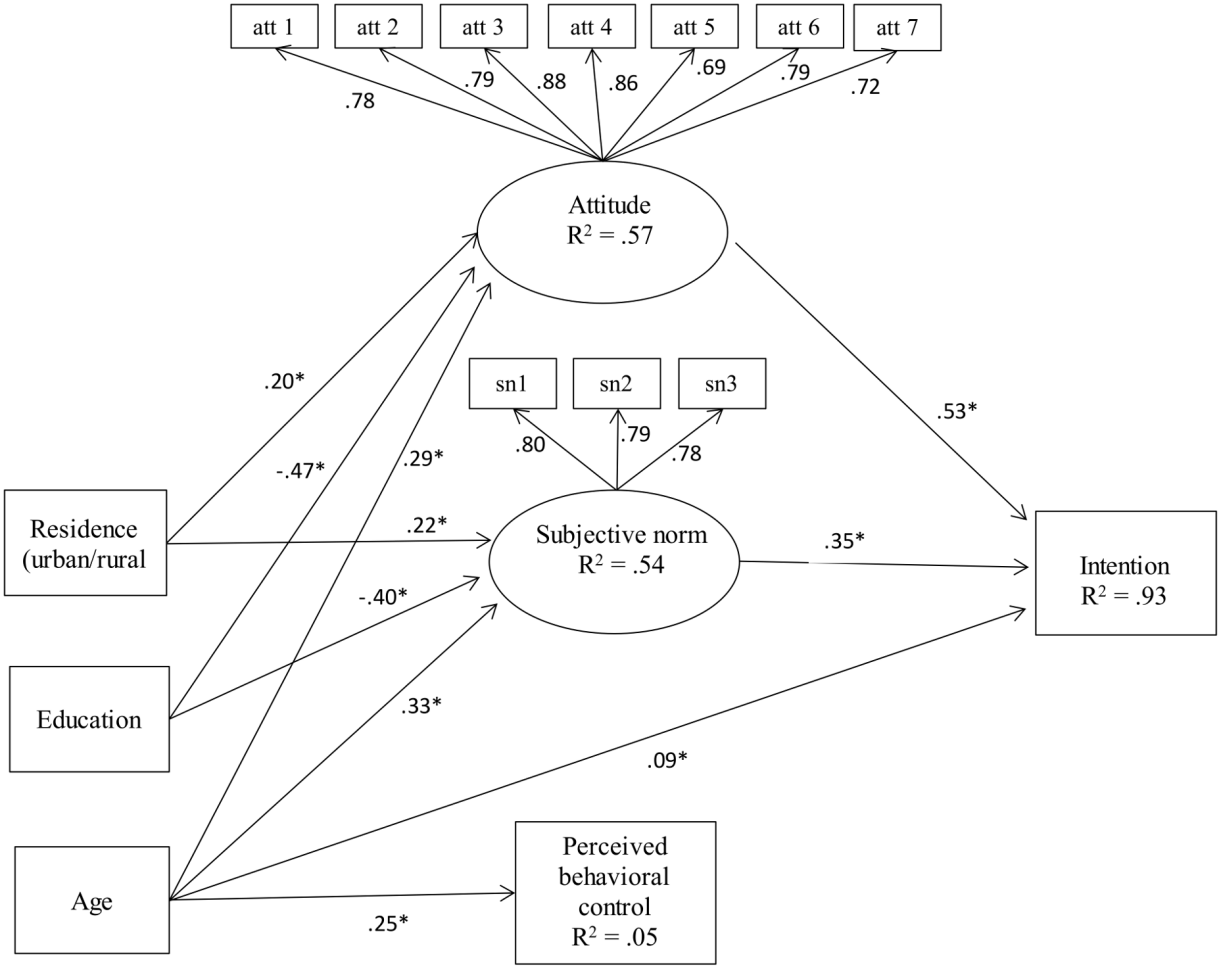
Structural model for the determinants predicting mothers’ intentions to allow their daughters to undergo FGM.

As shown in Fig 1, the measurement and structural model provided an adequate fit to the data. All factor loadings of the latent constructs were significant and above .69. Our analyses revealed that the study variables, together with the covariates, explained 93% of the total variance of the mothers’ intentions to mutilate their daughters. The attitudes (H1) and subjective norms (H2) were significantly related to the mothers’ intentions to mutilate their daughters. Attitudes had the strongest relationship with intention (β = .53, *p* <. 001), followed by subjective norms (β = .35, *p* <. 001). Thus mothers with a more favorable attitude and who perceived more social pressure from important others in their lives were more likely to have the intention to mutilate their daughters. Perceived behavioral control (H3) was not significantly related to intention (β = .03, *p* = .23).

With regard to the covariates, the residence of the mothers was significantly related to attitudes (β = .20, *p* <. 001) and subjective norms (β = .22, *p* <. 001), suggesting that mothers who live in a rural area had a more positive attitude toward female genital mutilation and perceived more social pressure from important others to mutilate their daughters. Furthermore, the education of the mothers was negatively related to attitudes toward female genital mutilation (β = -.47, *p* <. 001) and subjective norms (β = -.40, *p* <. 001). This suggests that higher educated mothers had less positive attitudes toward genital mutilation and perceived less social pressure to make their daughters undergo FGM. Finally, the mothers’ age was significantly related to attitudes (β = .29, *p* <. 001), subjective norms (β = .33, *p* <. 001), perceived behavioral control (β = .25, *p* <. 01) and intention (β = .09, *p* <. 001). Thus, older mothers had more positive attitudes toward female genital mutilation, felt more social pressure to allow their daughters to undergo FGM, perceived more control over their behavior and had a greater intention to make their daughters undergo FGM.

## Discussion

Our descriptive results indicate that 32.2% of the mothers intended to allow their daughters to undergo FGM. This finding is consistent with a study conducted by Yasin and colleagues in Iraqi Kurdistan, which investigated a sample with the same cultural tradition as our sample and found that 35% of the participants intended to mutilate their daughters [45]. From the relative weights of the structural model paths, we can conclude that mothers’ attitudes are the strongest predictor of their intention to allow their daughters to undergo FGM, suggesting that mothers with a more favorable attitude toward FGM are more likely to show the intention of mutilating their daughters. As such, health interventions should focus primarily on changing the attitudes and beliefs of the mothers. To date, in families in which FGM is common, girls who are not genital mutilated are often called rude girls and are believed to be promiscuous [46]. As described by Shell-Duncan and Hernlund [47], Gambian girls who are not mutilated are even called “Solema,” which is a highly derogatory term that refers to being “impolite, immodest, childish, unattractive, guilty and impure.” This goes to show that there is a long way to go before people’s attitudes are changed for good. It is therefore vital that the opinions and attitudes of mothers who intend to allow their daughters to undergo FGM are heard and understood by health care workers, so that they can adequately inform mothers about the adverse consequences of FGM.

Besides attitudes toward FGM, mothers’ subjective norms were strongly associated with respondents’ intentions to allow their daughters to undergo FGM. This finding is consistent with a study conducted by UNICEF [48], which found that the social pressure and moral judgments associated with FGM were key determinants of both the persistence and the rejection of the practice [48]. In Iran, it is the responsibility of health care workers, especially those working in health care centers, to consult mothers and to help them overcome feelings such as stress and fear, which may jeopardize their health or that of their children. Iranian health care workers directly communicate with mothers and their daughters, and most often are the first people to inform mothers of the mental and physical problems associated with FGM. Therefore, Iranian health care workers might be ideally suited to alleviate the social pressure of family members, husbands and neighbors. Unlike traditional midwives, Iranian health care workers are educated in the risks involved with genital mutilation. Although they have grown up in similar conditions to other Iranian men or women, their professional ethics might prevent them from having positive attitudes toward FGM, even when this conflicts with the beliefs or rules prescribed during their youth. Taking this into account, Iranian health care workers can have an important role to play both in the prevention of FGM in girls at risk and in the promotion of health and the treatment of women who have undergone this practice.

Although the findings suggest that mothers care about the perceived expectations of family members, neighbors and their husbands, we did not ask the mothers explicitly which of these people they considered to be most important. The findings of the above-mentioned UNICEF study, however, revealed that it is mothers, grandmothers and older female relatives who influence the decision to perform FGM the most. Mothers also reported that they would be blamed if they did not follow the customs [10,49] and that they feared social sanctions if they neglected the social norms [48]. Furthermore, many mothers believe that FGM can secure their daughters against social and cultural troubles. In other words, they believe that non-mutilated daughters will fall prey to social problems. This belief goes beyond the physical or mental dangers involved with FGM [8]. For practitioners, it might be useful to tell mothers that there are alternative ways to rid themselves of the guilt of not mutilating their daughters. For instance, some mothers already perform female genital mutilation symbolically by cutting a knife through the clothes of the daughter, instead of actually performing the mutilation [50].

In this study, we found no significant relationship between mothers’ perceived behavioral control and intention to allow their daughters to undergo FGM. This is not surprising, considering the fact that women in the selected regions do not enjoy great autonomy and lack empowerment. Ravansar is a region located in Kermanshah province, which is known as an undeveloped and poor province. Most women in Ravansar depend on their family because of their illiteracy and unemployment. Furthermore, women are often expected to obey their husbands, fathers and mothers in law [12]. As suggested by Shell-Duncan and colleagues [26], being genitally mutilated might serve as a signal to other genitally mutilated women that a girl or woman has been trained to respect the authority of her genitally mutilated elders [26].

With regard to the covariates, our study demonstrated that older mothers exhibited a greater intention to make their daughters undergo FGM compared to younger respondents. In a way, this finding suggests that FGM will be reduced in the future because the new generations of mothers are less inclined to believe that their daughters should undergo female genital mutilation. Although this might indicate a loosening of the tradition in Iran, it is important to note that Masho and Matthews [4] pointed out that younger woman are still likely to continue the practice. We also found that less educated mothers have more positive attitudes toward FGM, which is consistent with the majority of studies that have investigated the association between education and FGM [12,20,51,52]. The results of our study further indicated that women who resided in an urban region had less positive attitudes toward FGM and felt less social pressure to engage in the behavior. These findings are in line with other studies [4,52,53]. One possible explanation for this finding is that urban residents have more access to the mass media and to education [54] and, as such, might be more aware of the negative consequences of FGM for the mental and physical health of their daughters.

The present study has several limitations. One limitation is sampling bias, which may limit the external validity of the study findings. This study was conducted in Ravansar, a county in western Iran, in which nearly everyone has a Kurdish background. Since the determinants of FGM might differ from region to region and FGM might be related to ethnicity, the generalizability of the findings is limited. Furthermore, we only recruited mothers who had medical records at a health center and had experienced female genital mutilation. Alternative participant recruitment and data collection strategies may be needed to minimize sampling bias in future studies, and corroboration of our findings by data gathered in other regions where FGM is common practice would lend credibility to the findings. Second, the survey data are cross-sectional in nature, which means that inferences with respect to causality should be made with caution. We assume directionality in the relationships between the variables in the model (e.g., the influence of subjective norms on intention), but we acknowledge that causality can only be inferred theoretically. However, the use of theory and prior research that guides this study lends support to the inferences. In future studies, a longitudinal or experimental research design could be deployed to provide further support to the directionality of the observed relationships in our study. Third, the data collection was based on self-report measures. Self-reports may be subject to response distortions (e.g., extreme- or central-tendency responding, negative affectivity bias, socially desirable responding), which might inflate the associations between independent and outcome variables [55,56]. In addition, although the midwives were trained to help illiterate mothers fill out the questionnaires, their help might have resulted, to some extent, in social desirability bias. Fourth, we did not investigate how deeply religious the mothers were. In addition, the present study did not capture several background characteristics such as the age of the daughters, the total number of children and the living situation (e.g., living with a mother-in-law). It would be interesting if future research could verify how the degree of religiosity might influence the intention to allow daughters to undergo FGM; this is because it has been demonstrated that some people believe that FGM must be done for religious reasons [57]. It would also be interesting if future research could explore how other background characteristics (e.g., the number of daughters a mother has) might influence the mothers’ intentions to allow their daughters to undergo FGM.

## Conclusion

Despite the severe negative effects of female genital mutilation (FGM) on the mental and physical health of women, it is still a common practice in Ravansar, a county in western Iran. To prevent this extremely harmful practice, it is essential to understand and change the motivations to practice it, especially those motivations that relate to mothers’ intentions to perform the practice. Therefore, the aim of this study was to examine factors that influence mothers’ intentions to make their daughters undergo FGM. To investigate the determinants that influence this intention, the current research tested a model based on the theory of planned behavior conducted with a paper-and-pencil survey of 300 mothers living in Ravansar. We found that attitudes, subjective norms and age significantly predicted the intention of mothers to allow their daughters to undergo FGM, and that education, residence and age significantly predicted mothers’ attitudes and subjective norms. Evidence has shown that education and health promotion can encourage mothers to stop performing FGM. Therefore, a significant change in factors such as economic development, literacy, education, health promotion and social development might cause a gradual decline in FGM behavior.

## Supporting Information

S1 Data (SAV)

S1 Questionnaire (DOCX)
