# Supplementary material for: Daughters at Risk of Female Genital Mutilation: Examining the Determinants of Mothers’ Intentions to Allow Their Daughters to Undergo Female Genital Mutilation
Source: PLoS One. 2016 Mar 31;11(3):e0151630. doi: 10.1371/journal.pone.0151630 (PMC4816284; doi:10.1371/journal.pone.0151630)
Supplement: S1 Questionnaire — (DOCX) [file pone.0151630.s002.docx]

|  | *strongly disagree* | Disagree | Neither agree nor disagree | Agree | *strongly agree* |
| --- | --- | --- | --- | --- | --- |
| Att1. Female genital mutilation is a good tradition |  |  |  |  |  |
| Att2. Female circumcision is good for controlling female sexuality |  |  |  |  |  |
| Att3. Circumcision is performed for religious reasons |  |  |  |  |  |
| Att4. Female genital mutilation increases women’s health |  |  |  |  |  |
| Att5. Female genital mutilation is a violent behavior (reverse scored) |  |  |  |  |  |
| Att6. Female genital mutilation should continue |  |  |  |  |  |
| Att7. Female circumcision increases the chances of marriage |  |  |  |  |  |
| **Subjective norm** |  |  |  |  |  |
| Sn1. Family members expect me to make my daughter(s) undergo FGM |  |  |  |  |  |
| Sn2. Neighbors put me under pressure make my daughter(s) undergo FGM |  |  |  |  |  |
| Sn3. My husband wants me to make my daughter(s) undergo FGM |  |  |  |  |  |
| **Perceived behavioral control** |  |  |  |  |  |
| Despite the difficulties, I can prevent my daughter(s) from being circumcised |  |  |  |  |  |

**Intention:**

“Do you intend to make your daughter(s) undergo female genital mutilation? Yes No
